# Interaction of different forms of graphene with chicken embryo red blood cells

**DOI:** 10.1007/s11356-017-9788-5

**Published:** 2017-07-28

**Authors:** Sławomir Jaworski, Mateusz Hinzmann, Ewa Sawosz, Marta Grodzik, Marta Kutwin, Mateusz Wierzbicki, Barbara Strojny, Krishna P. Vadalasetty, Ludwika Lipińska, André Chwalibog

**Affiliations:** 1 Faculty of Animal Science, Department of Animal Nutrition and Biotechnology, Warsaw University of Life Sciences, Ciszewskiego 8, 02-786 Warsaw, Poland; 2 Department of Veterinary Clinical and Animal Sciences, University of Copenhagen, Copenhagen, Denmark; 3 Institute of Electronic Materials Technology, Warsaw, Poland

**Keywords:** Graphene, Red blood cells, Toxicity

## Abstract

With the rapid development of graphene synthesis and functionalization approaches, graphene and its related derivatives have shown great potential in many applications in material science, including biomedical applications. Several in vitro and in vivo studies clearly showed no definitive risks, while others have indicated that graphene might become health hazards. In this study, we explore the biocompatibility of graphene-related materials with chicken embryo red blood cells (RBC). The hemolysis assay was employed to evaluate the in vitro blood compatibility of reduced graphene, graphene oxide, and reduced graphene oxide, because these materials have recently been used for biomedical applications, including injectable graphene-related particles. This study investigated structural damage, ROS production and hemolysis of chicken embryo red blood cells. Different forms of graphene, when incubated with chicken embryo RBC, were harmful to cell structure and induced hemolysis.

## Introduction

Graphene, a newly discovered allotrope of carbon, is a single, two-dimensional layer of carbon atoms (Novoselov et al. 2004). Carbon atoms, through hybridization between one σ orbital and two π orbitals, form trigonal planar structures with σ bonds between them and π bonds perpendicular to the planar structure. Since its discovery, graphene has attracted great attention in the fields of biology and medicine, including areas such as drug/gene delivery (Feng et al. 2011; Liu et al. 2008 and Wu et al. 2014), cancer therapy (Zhang et al. 2010), bioimaging (Shen et al. 2012), biosensing (Shen et al. 2012), antibacterial materials (Hu et al. 2010), and tissue scaffolds (Nayak et al. 2011). Despite these promising applications, the potential toxicity of graphene, although widely investigated, is still unclear. Several studies indicate graphene properties which might be related to its toxicity, such as surface area (Mu et al. 2012; Akhavan et al. 2012), number of layers (Sanchez et al. 2012), lateral dimension (Yue et al. 2012), surface chemistry (Wang et al. 2013), and purity/method of production (Park and Ruoff 2009; Du et al. 2013; Ciesielski and Samori 2013). A study by Chang et al. (2011) has shown that graphene oxide (GO) had no significant cytotoxic effect on A549 cells. However, Wang et al. (2013) reported that modifications of GO, resulting in its surface change, had a toxic effect on human lung fibroblasts. Recent studies conducted by our team indicate that pristine graphene (GN) causes both genotoxic and dose-dependent cytotoxic effects towards the U87 cell line. Genotoxic effects were also caused by reduced graphene oxide (rGO), but not by GO (Jaworski et al. 2013; Hinzman et al. 2014). GN is able to induce apoptosis in macrophages (Li et al. 2012), while GO, after intravenous administration in mice, caused cell infiltration, inflammation, and other pathological changes (Zhang et al. 2011).

Most of the drug/gene delivery platforms require intravenous administration; thus, proper assessment of potential toxic effects of carriers in such systems is of utmost importance. A study conducted by Sasidharan et al. (2012) showed that pristine and functionalized graphene exhibit a very high hemocompatibility, causing less than 0.2% of hemolysis in RBC in concentrations up to 75 μg/ml. Another study indicated that graphene oxide and graphene sheets (GS) exhibited dose-dependent hemolysis. The extension of hemolysis depended on the size of used nanoparticles and on their oxygen content (Liao et al. 2011). However, due to a very high diversity of graphene family materials, the knowledge about their interaction with blood cells is still insufficient. Thus, we report our findings regarding interactions between red blood cells (RBC) and GN produced by physical exfoliation and GO and rGO synthetized by chemical processes. We hypothesized that due to different properties of used graphene, emerging from different methods of its production, the toxic effects exerted towards RBC may differ. Therefore, the objective of this study was to compare toxic effects of different forms of graphene on RBC.

## Material and methods

### Preparation and characterization of graphene platelets

Graphene powders (purity >99.99%) were purchased from the following providers: GN from SkySpring Nanomaterials (Houston, TX, USA) and GO and rGO from the Institute of Electronic Materials Technology (Warsaw, Poland). GN was produced by liquid-phase exfoliation of graphite, whereas GO was produced by chemical oxidation of graphite, and rGO by chemical reduction of GO. GO was prepared by a modified Hummer’s method from acid-washed graphite platelets: 5 g of graphite was added to 125 ml of sulfuric acid, and 3.25 g of potassium nitrate was added before the start of the reaction. The mixture was stirred with a mechanical stirrer. Subsequently, the beaker with reagents was kept below 5 °C in a water/ice bath while 15 g of potassium permanganate was gradually added. The beaker was taken out of the bath and kept at 30–35 °C with continuous stirring then left at the room temperature. In the next step, deionized water was added to the stirred mixture so that the temperature did not exceed 35 °C. The beaker was put into a water bath at a temperature of 35 °C and stirred for another 1 h. The constantly stirred mixture was then heated to 95 °C for 15 min. To stop the reaction, 280 ml of deionized water and 5 ml of hydrogen peroxide were added. The mixture was rinsed with hydrogen chloride solution to remove sulfate ions and then rinsed with deionized water to remove chloride ions. To prepare the rGO, a water suspension of 50 mg of graphene oxide was acidified to pH = 1 and heated to 90 °C. Then 12 ml of reducing mixture (0.01 g of ammonium iodide, 9 g of hydrated sodium hypophosphite, and 1.21 g of sodium sulfite dissolved in 100 ml of deionized water) was added. A black material (rGO) immediately precipitated. The product was filtered, washed with deionized water, and dried.

Shape and size of the graphene nanoplatelets were evaluated using a JEM-2000EX transmission electron microscope (TEM) at 80 keV (JEOL Ltd., Tokyo, Japan) and FEI QUANTANA 200 scanning electron microscope (SEM). The samples for TEM were prepared by placing hydrocolloid droplets into Formvar-coated copper grids (Agar Scientific, Stansted, UK). The test was performed in triplicate. Zeta potential was measured in milli-Q water by a ZEN3500 Zetasizer Nano ZS (Malvern Instruments, Malvern, UK). The content of chemical bonds was identified from Fourier transform infrared (FTIR) spectra and recorded on a Tensor 27 FTIR spectrometer (Bruker, Billerica, MA, USA), with 32 scans at a resolution of 2 cm^−1^ in the frequency range 650–4000 cm^−1^. FTIR direct-transmittance spectroscopy (KBr) was used to indicate the degree to which oxygen groups were removed, and the IR absorption of water from the air was mostly eliminated. Graphene samples were measured as a pastille mixed with KBr and compacted under high pressure (>10× atmospheric pressure). Each measurement was recorded immediately and repeated three times.

Prior to application, the carbon nanoparticles were dispersed in phosphate buffered saline (PBS) to prepare the following concentrations: 50, 500, and 5000 μg/ml. The solutions were then sonicated for 30 min.

### Embryo model

Fertilized eggs (*Gallus gallus*, *n* = 15) from Hubbard Flex Line hens were obtained from a commercial hatchery (Dembowka, Poland). After 19 days of egg incubation (temperature 37 °C, 70% humidity, turning once per hour), the embryos were immediately decapitated while blood samples were collected from the jugular vein. Blood samples were divided into the following groups: control untreated (0% hemolysis), positive control treated with 3% hydrogen peroxide (100% hemolysis), GN 50 μg/ml, GN 500 μg/ml, GN 5000 μg/ml, GO 50 μg/ml, GO 500 μg/ml, GO 5000 μg/ml, rGO 50 μg/ml, rGO 500 μg/ml, and rGO 5000 μg/ml hydrocolloids diluted in PBS. The samples were placed in Vacutainer tubes (BD Inc., Franklin Lakes, NJ, USA) containing ethylenediaminetetraacetic acid (EDTA), gently mixed on a rotary shaker, and incubated for 3 h at 37 °C. The incubation time was based on Kutwin et al. (2014). All measurements were performed with three replicates.

### Blood cell morphology

Blood cell morphology was investigated using light microscopy, SEM, and TEM.

Peripheral blood smears were prepared using 5 μl of whole blood, air-dried, stained peripherally with May-Grünwald-Giemsa, and examined at a magnification of ×1.000 (Leica DM750, Leica Microsystems, Nussloch, Germany).

For the SEM examination, the blood samples were centrifuged for 5 min at 1200 rpm (Sorvall ST 16, Termofisher Scientific, Waltham, USA). The RBC pellet was washed in PBS (0.01 M, pH 7.2; P4417, Sigma), fixed in 2.5% glutaraldehyde (G5882, Sigma) for 1 h, washed twice in PBS, and placed on aluminum SEM stubs. The SEM stubs were kept in a moist atmosphere for 1 h, washed in PBS, post-fixed in 1% osmium tetroxide (75,632, Sigma) for 1 h, rinsed in distilled water, and dehydrated with progressive alcohol solutions (30–50–70–90–95%) and finally twice in absolute alcohol. The preparations were further dehydrated with a critical point-dried (Polaron CPD 7501, Quorum Technologies, Newhaven, East Sussex, UK) and covered by a thin layer of gold (JEE-4C, JEOL Ltd., Tokyo, Japan). The samples were inspected by SEM at 1 KeV (FEI Quanta 200, FEI Co., Hillsboro, OR, USA).

For the TEM examination, the blood samples were fixed in 2.5% glutaraldehyde and centrifuged at 1200 rpm. The supernatant was discarded; RBCs were dispersed in deionized water. The samples for TEM were prepared by placing hydrocolloid droplets into Formvar-coated copper grids. The test was performed in triplicate.

### Hemolytic assay

The hemolysis assay was performed with embryo whole blood. The cells are centrifuged for 10 min at 1200 rpm, and the absorbance of the supernatant, which includes plasma and lysed erythrocytes, was measured at 540 nm (Infinite M200, Tecan, Durham, NC, USA). Percentage of cell lysis was determined as compared to the positive group (100% of hemolysis according to Kutwin et al. (2014).

### ROS production

The measurement of reactive oxygen species (ROS) production was performed with embryo whole blood. The samples (untreated control, positive control treated with 3% hydrogen peroxide, GN 50 μg/ml, GN 500 μg/ml, GN 5000 μg/ml, GO 50 μg/ml, GO 500 μg/ml, GO 5000 μg/ml, rGO 50 μg/ml, rGO 500 μg/ml, and rGO 5000 μg/ml) were placed in Vacutainer tubes containing EDTA, gently mixed on a rotary shaker, and incubated for 3 h at 37 °C. ROS production was evaluated using DCFDA—Cellular Reactive Oxygen Species Detection Assay Kit (Abcam, Cambridge, UK). One hundred microliter of each sample was transferred to opaque-bottomed 96-well plates, and 100 μl of diluted DCFDA was added to each well and incubated for an additional 30 min at 37 °C in the dark. ROS production was measured by fluorescence spectroscopy with excitation wavelength at 485 nm and emission wavelength at 535 nm on an enzyme-linked immunosorbent assay reader (Infinite M200, Tecan, Durham, NC, USA).

### Statistical analysis

Statgraphics Centurion software (StatPoint Technologies, Warrenton, VA, USA) was used for the statistical analysis. The data were analyzed using multifactorial analysis of variance (ANOVA) followed by Tukey’s multiple range test. *P* values <0.05 were considered to be statistically significant.

## Results

### Characterization of graphene nanoplatelets

**Fig. 1:**
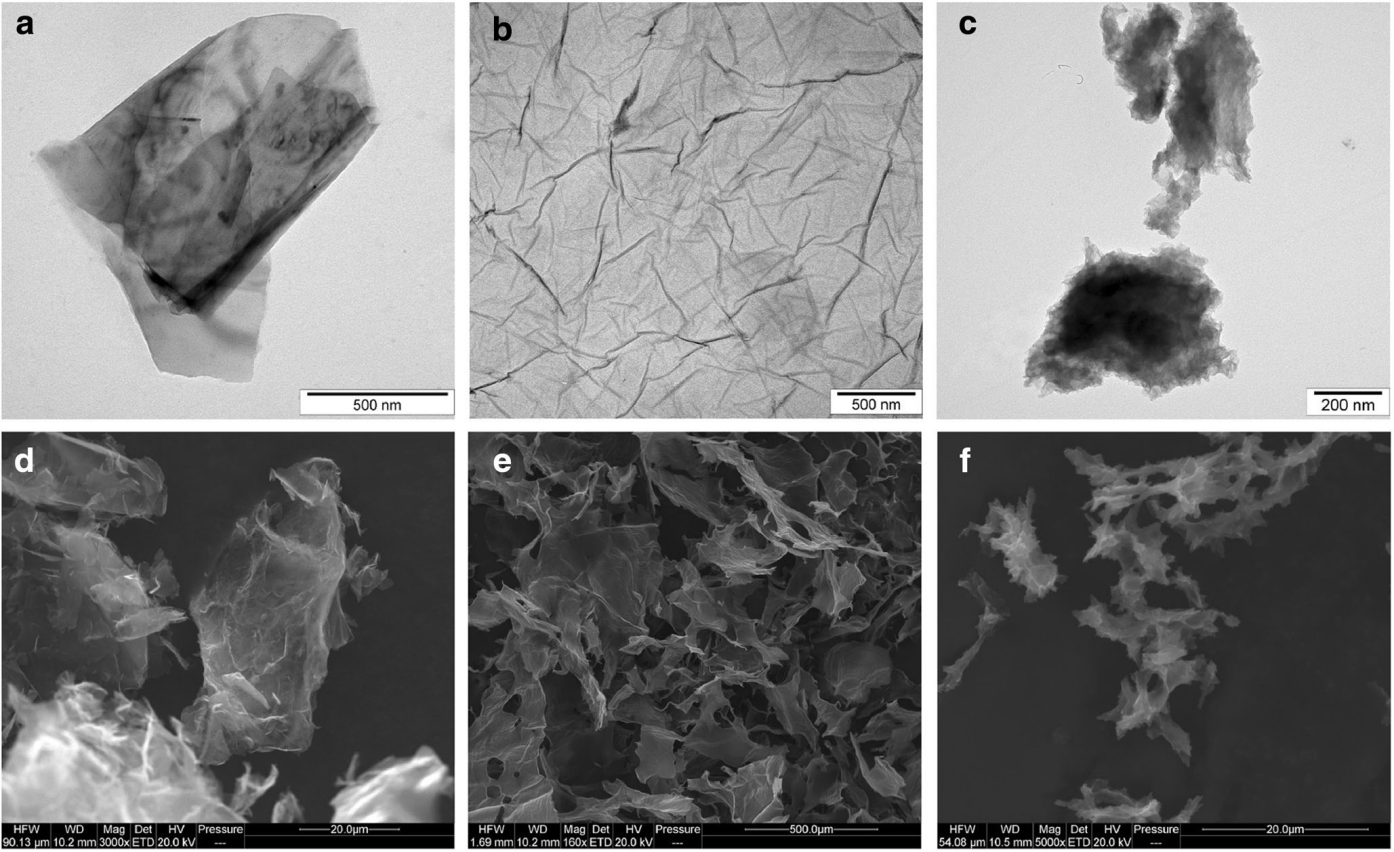
Characterization of pristine graphene (**a**, **d**), oxidized graphene (**b**, **e**), and reduced oxidized graphene (**c**, **f**) by transmission electron microscopy (**a**, **b**, **c**) and scanning electron microscopy (**d**, **e**, **f**)

**Fig. 2:**
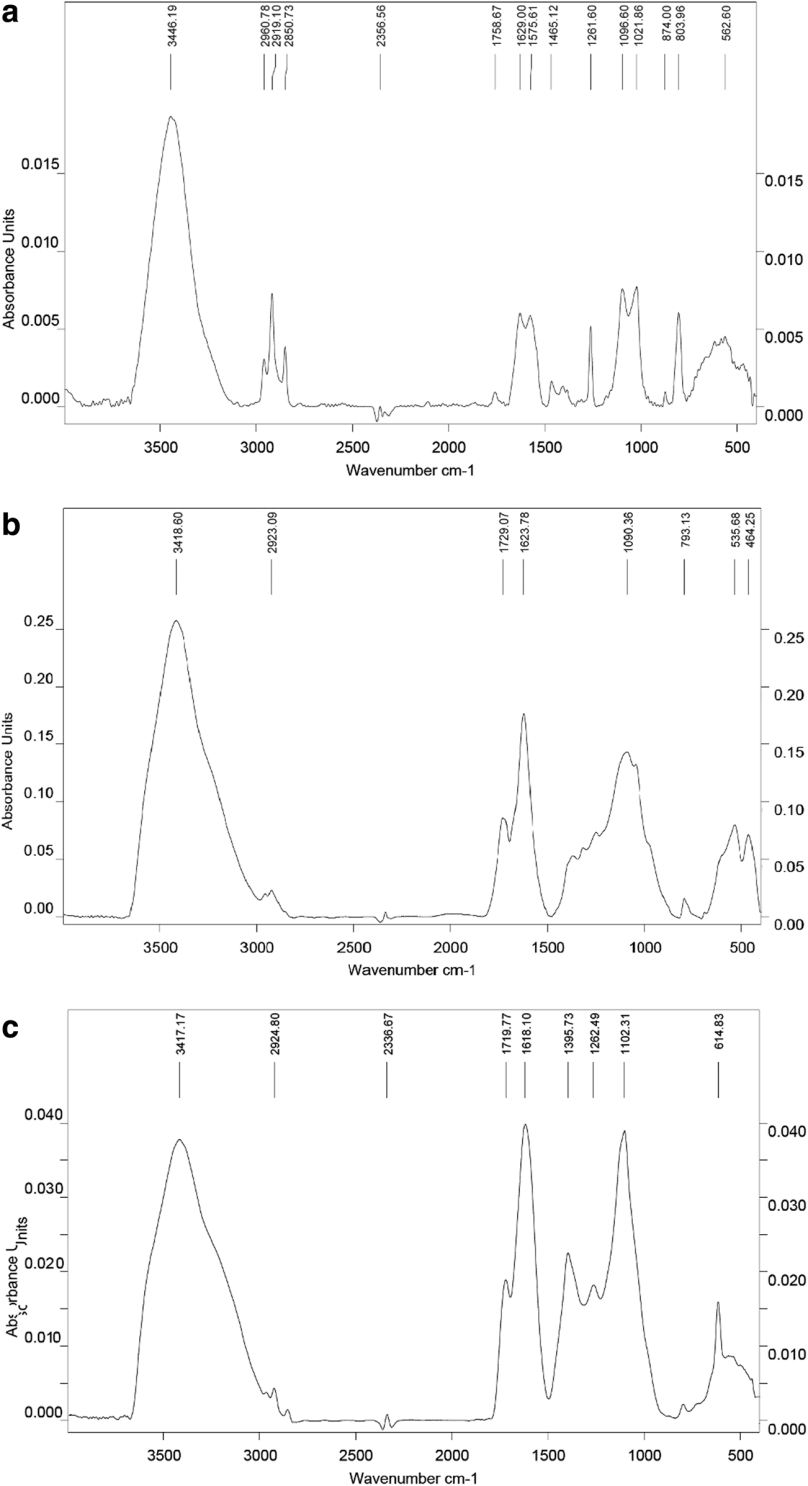
Room-temperature Fourier transform infrared spectra of pristine graphene (**a**), oxidized graphene (**b**), and reduced oxidized graphene (**c**)

The mean Zeta potential for the nanoparticle samples was −8.52 for GN, −43.2 for GO, and −29.6 for rGO. Figure 1 shows representative TEM and SEM images of the graphene platelets. Most of the graphene platelets were visible as a single layer or a few layers. The shape of GN, GO, and rGO platelets was irregular with jagged edges. Hydrophilic GO platelets formed a single layer, while hydrophobic GN and rGO platelets often created agglomerates. The thickness of the materials used was in the nanoscale range, but the surface area was not, ranging from 100 nm to 10 μm and forming agglomerates over 10 μm in diameter. The surface diameter of the GN ranged from 400 nm to 10 μm. GO platelets ranged from 100 nm to 2.3 μm. The rGO platelets were smaller, ranging from 100 nm to 1.5 μm in diameter, but agglomerates were more than 5 μm in diameter. Figure 2 shows typical FTIR spectra obtained for the different forms of graphene in this study. GN had the main peak for C=C bonds at 1572 cm^−1^. GN also had peaks at 1720–1757 cm^−1^, due to C=O stretching vibrations from carbonyl and carboxylic groups. The most characteristic feature for all GO and rGO platelets was the broad, intense band at 3430–3444 cm^−1^, which can be attributed to the O–H stretching vibrations of hydroxyl groups in adsorbed water molecules, structural OH groups, and carboxylic acids. GO and rGO also had peaks at 1239–1261 cm^−1^ caused by C–O–C stretching vibrations from epoxy-functional groups (Table 1).

**Table 1:** Comparison of the physical characteristics of pristine graphene (GN), oxidized graphene (GO), and reduced oxidized graphene (rGO)

Parameter	GN	GO	rGO
Shape	Irregular	Irregular, filmlike	Irregular
Zeta potential (mV)	−8.52	−4.32	−29.6
Size	400 nm–1.5 μm	100 nm–2.3 μm	100 nm–1.5 μm, aggregates >5 μm
Surface chemical bonds	C=C, C–O–C, O–H	C=C, C–O–C, O–H, C–O	C=C, C–O–C, O–H, C–O
Production	Exfoliation	Modified Hummer’s method	Chemical reduction of GO

### Microscopic evaluation of morphological changes

**Fig. 3:**
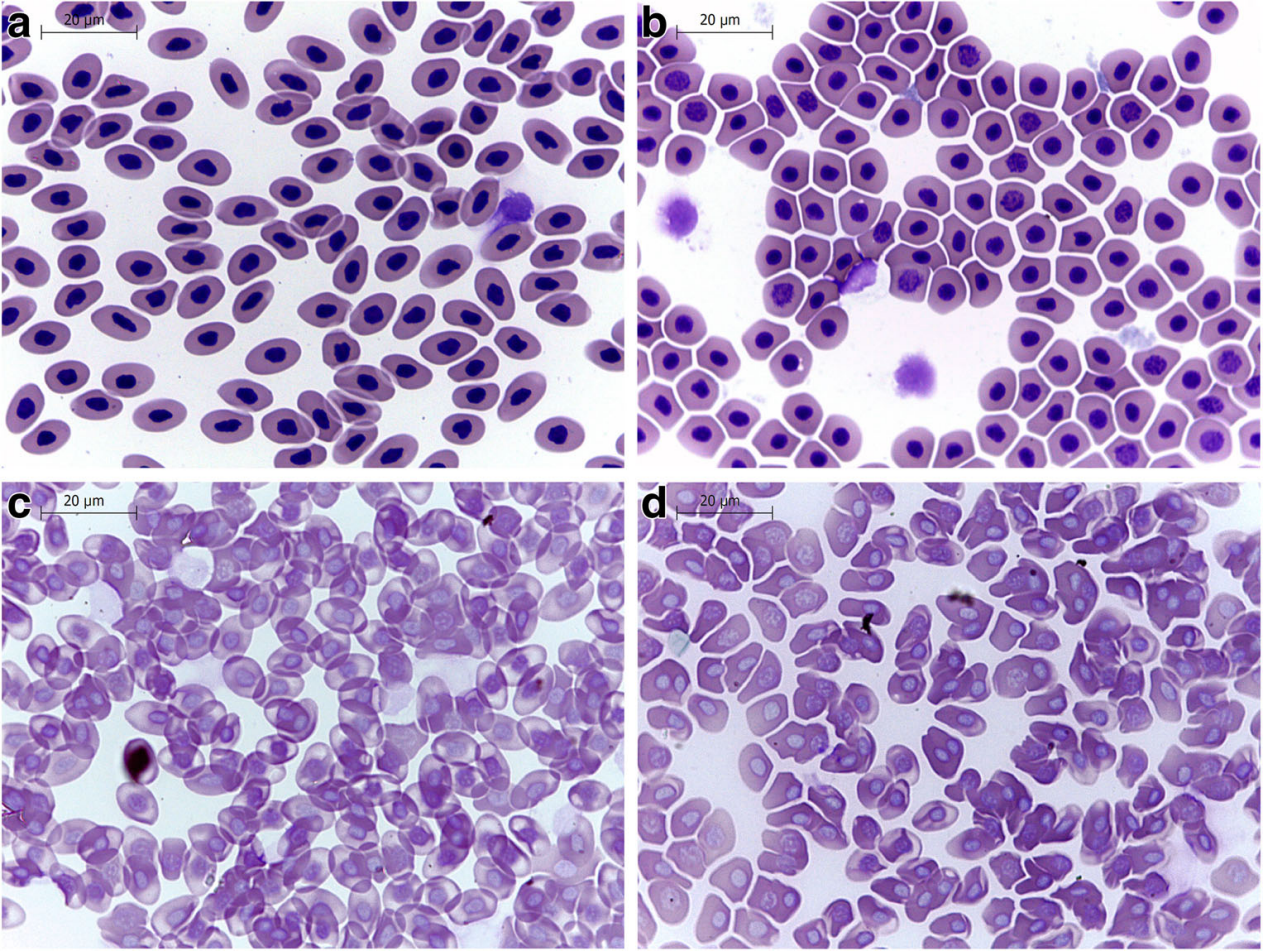
Red blood cell morphology by light microscopy. **a** Control (without treatment). **b** Pristine graphene. **c** Oxidized graphene—GO. **d** Reduced oxidized graphene—rGO. *Black arrows* point to GO agglomerates; *red arrows* point to rGO agglomerates

**Fig. 4:**
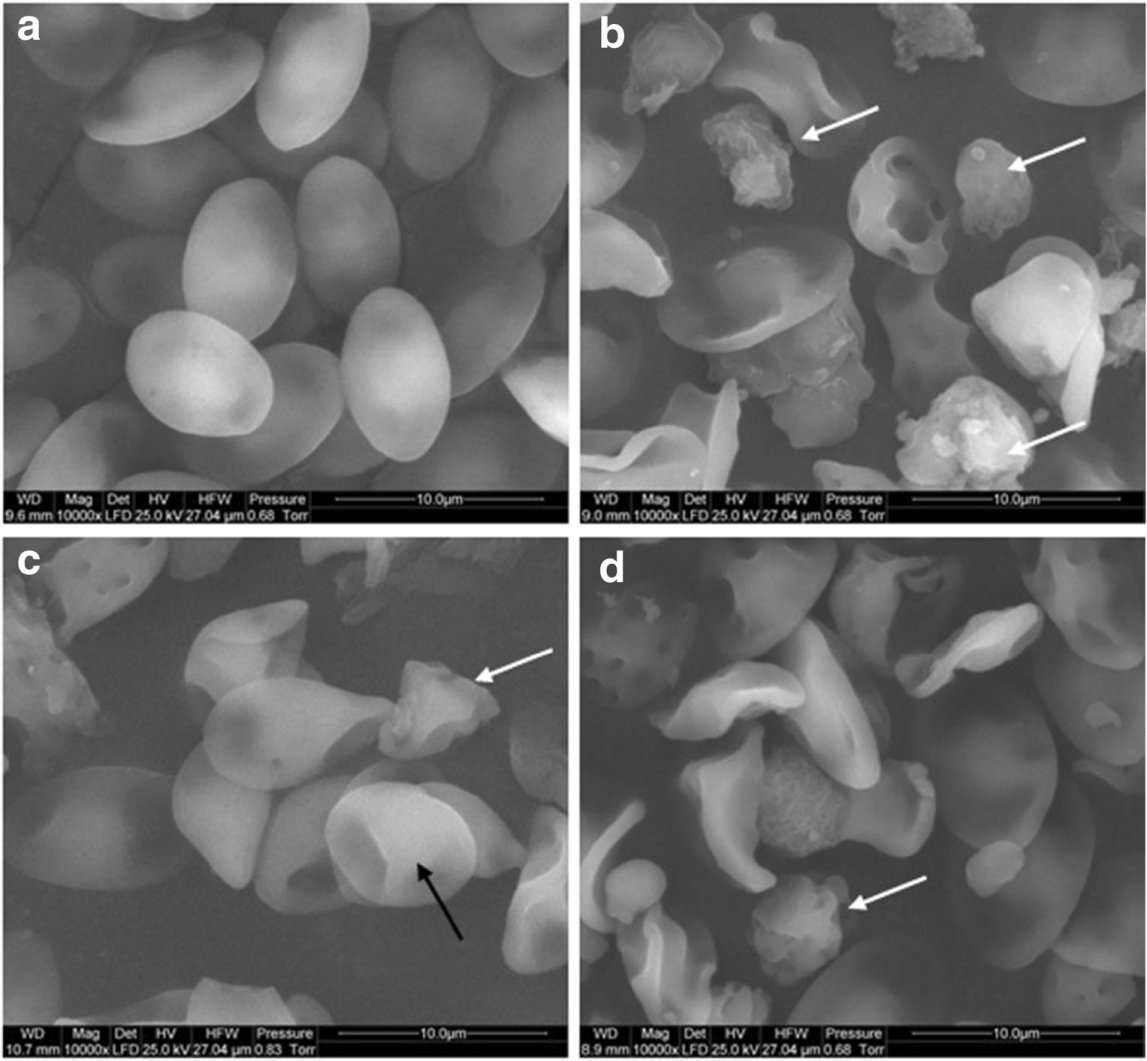
Visualization of red blood cell morphology by scanning electron microscopy. **a** Control (without treatment). **b** Pristine graphene. **c** Oxidized graphene. **d** Reduced oxidized graphene. *Black arrows* point to knizocyte; *white arrows* point to echinocyte. Scale bar, 10 μm

**Fig. 5:**
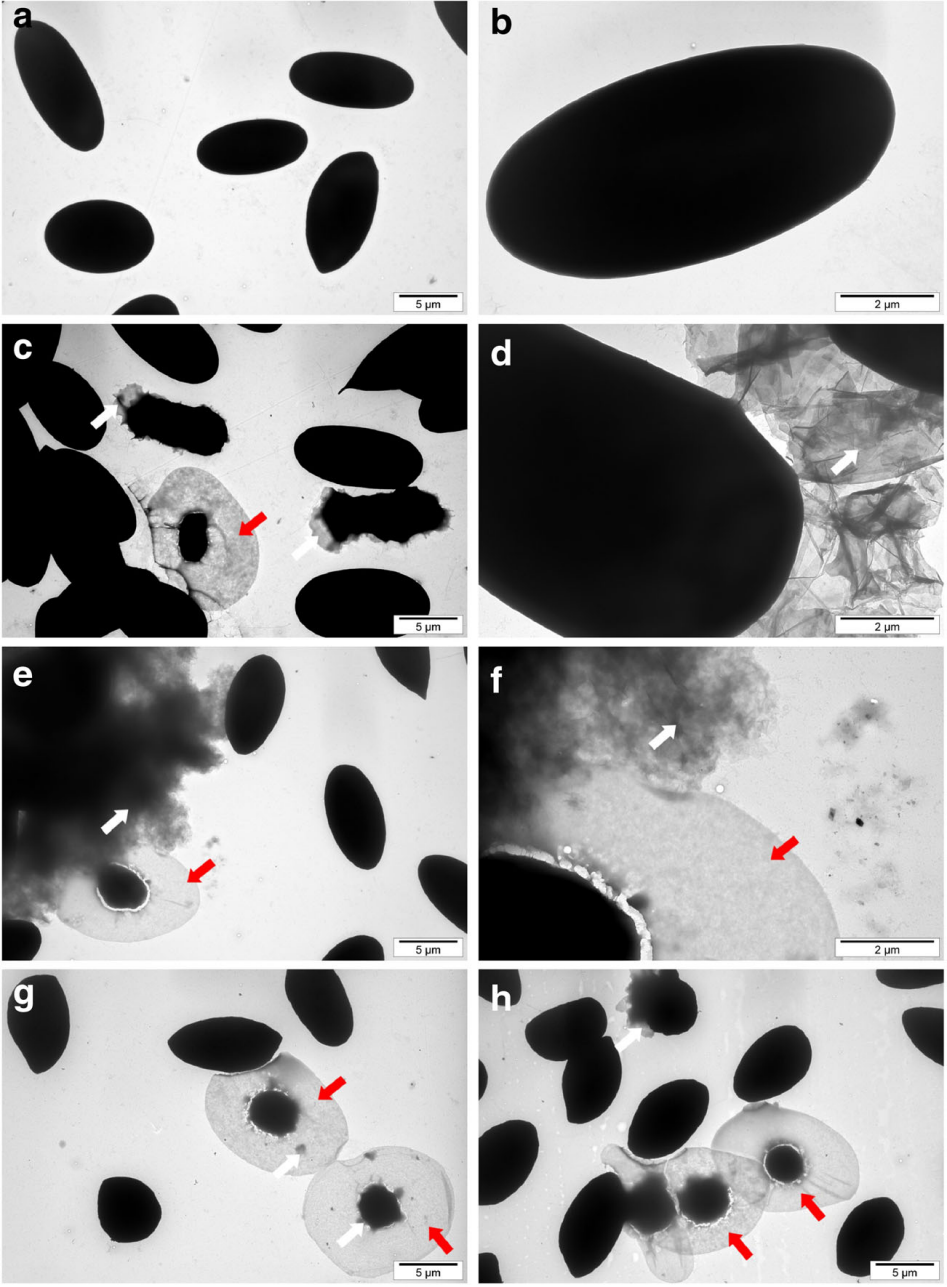
Visualization of red blood cell morphology by transmission electron microscopy. **a**, **b** Control (without treatment). **c**, **d** Pristine graphene. **e**, **f** Oxidized graphene. **g**, **h** Reduced oxidized graphene. *White arrows* point to graphene agglomerates; *red arrows* point to ghost cells. Scale bar, 5 μm (**a**, **c**, **e**, **g**, **h**) and 2 μm (**b**, **d**, **f**)

Morphological changes and significant lysis of RBC after GN, GO, and rGO exposure were observed by light microscopy, SEM, and TEM. Compared to the normal biconcave shape of untreated RBC in PBS, RBC treated with GN, GO, and rGO demonstrated both aberrant morphology and recently lysed RBCs (Fig. 3). The cell membranes were disintegrated; the shape of cells was deformed, but cells also lost their biconcavity. The observation also showed the increasing level of swollen cells as a result of hemolysis. Hemagglutination was also observed in all treated groups (Figs. 4 and 5). The SEM and TEM images showed cell membrane degradation and loss of the typical discoid-shape of cells. Pathological forms of erythrocytes, such as echinocytes and knizocytes, were observed in all treated groups. Analysis of TEM samples from all treated groups showed the presence of ghost cells, which are the result of cell lysis.

### Hemolysis

**Fig. 6:**
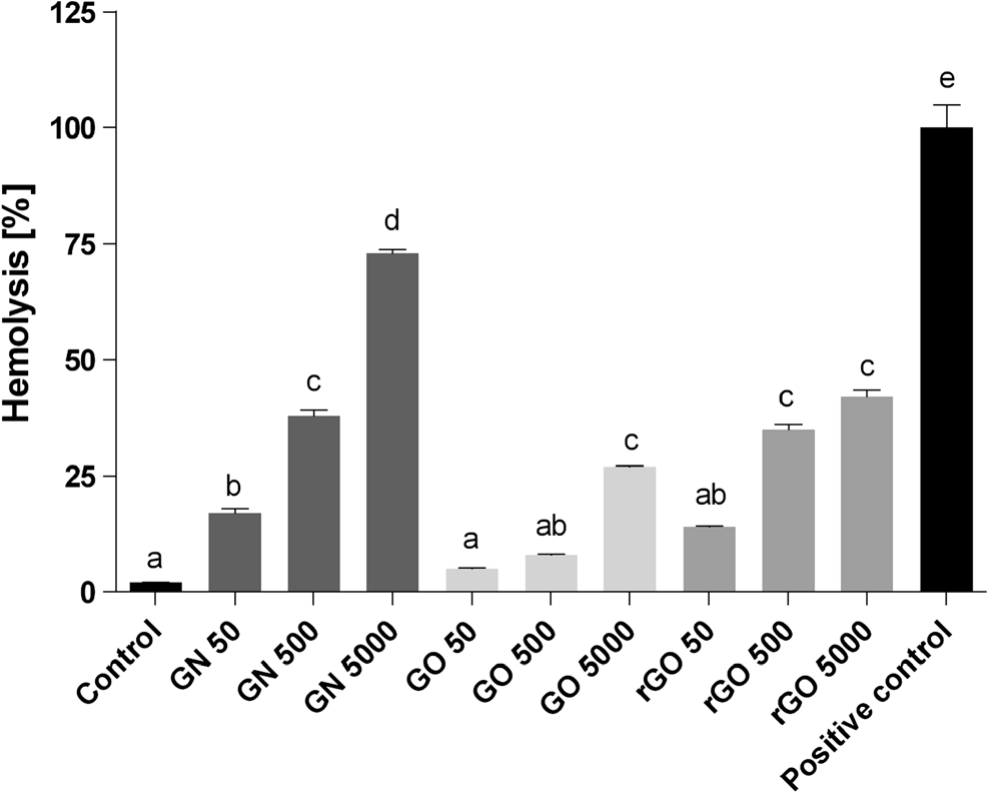
Level of hemolysis of red blood cells. Error bars indicate standard error of the mean. Bars with different superscripts denote statistically significant differences between the control group (non-treated) and groups treated with the following: pristine graphene, oxidized graphene, reduced oxidized graphene, and 3% hydrogen peroxide-positive control (*P* < 0.05). There were significant differences (*P <* 0.05) between different concentrations of graphenes

In contrast to the control group, GN, GO, and rGO showed hemolytic properties. Increased concentrations of GN, GO, and rGO resulted in increased hemolysis (Fig. 6). Furthermore, the percentage of hemolysis was the highest for GN—73% at a concentration of 5000 μg/ml. In GO- and rGO-treated samples, the highest hemolysis rates were observed at the same concentration—27 and 42%, respectively. The lack of hemolysis in the control group and an almost 100% hemolysis rate in the positive control group treated with 3% hydrogen peroxide confirmed the accuracy of the assay.

### ROS production

**Fig. 7:**
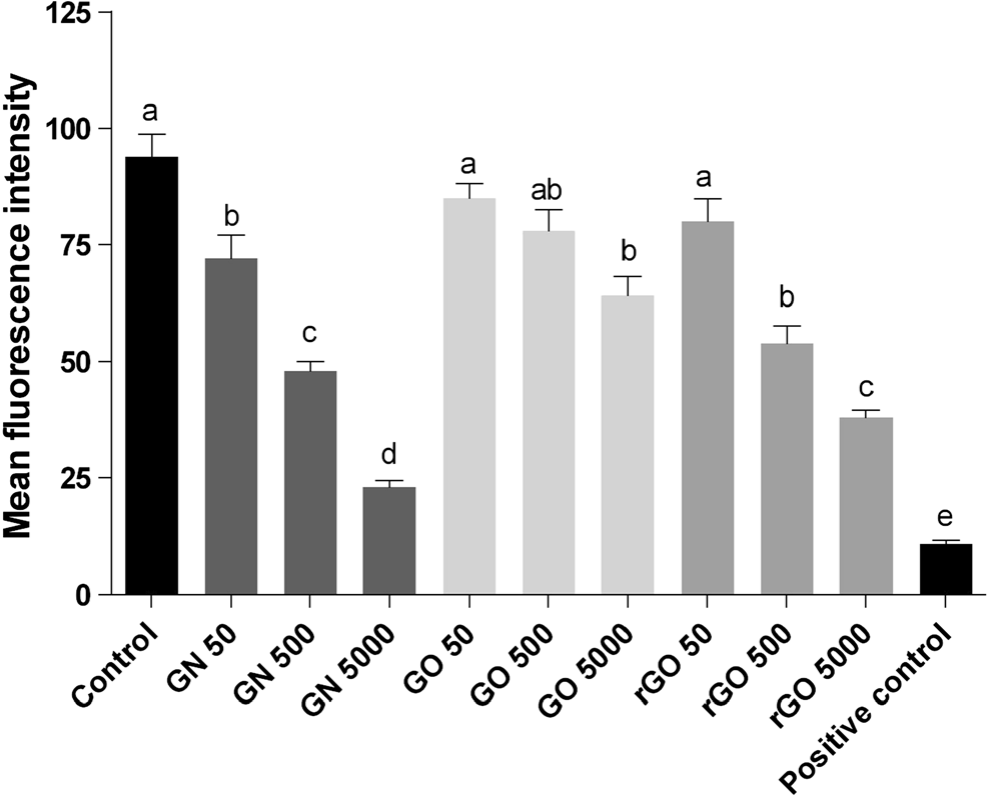
Effect of pristine graphene (GN), oxidized graphene (GO), and reduced oxidized graphene (rGO) on the ROS production of red blood cells. Bars with different superscripts denote statistically significant differences between the control group (non-treated) and groups treated with the following: GN, GO, rGO, and 3% hydrogen peroxide-positive control (*P* < 0.05). There were significant differences (*P <* 0.05) between different concentrations of graphenes

GN, GO, and rGO significantly (*P* < 0.05) increased the ROS production of RBC compared with the control group. Increased concentrations of all types of graphene resulted in increased ROS generation. The highest was observed at a concentration of 5000 μg/ml (Fig. 7).

## Discussion

In our study, we used red blood cells from the chicken embryo model, which allows not only a quick verification of the hemocompatibility, but also represents a good and precise model for the evaluation of toxicity (Rashidi and Sottile 2009). A recent study has shown that both surface chemistry and size of graphene platelets play a key role in the toxicity, distribution, and excretion of graphene; therefore, different graphene materials may have different influences on the organism (Yang et al. 2013). In the present experiment, the three different types of graphene nanoplatelets had adverse effects on RBC. Incubation with GN, GO, and rGO nanoparticles altered the RBC morphology and led to hemolysis. The cell membranes were disintegrated and their shape deformed. Moreover, cells lost their biconcavity. Although all of the graphene nanoplatelets exhibited dose-dependent hemolytic activity towards RBC, there were differences in the extent of hemolysis between different forms of graphene. GN had the highest hemolytic activity and GO the lowest. This fact may be explained by different chemical and physical properties of the investigated nanoparticles. GO nanoparticles are hydrophilic, well-dispersed in water, and form stable hydrocolloids. On the other hand, GN and rGO are hydrophobic, often creating agglomerates in water, which could then easily adhere to cell membranes. Due to the irregular edges of GN and rGO, this adhesion may result in mechanical disruption of RBC’s plasma membrane integrity, causing leakage. However, due to its hydrophilic properties, GO interacts with cell membranes in a different manner than GN and rGO and is less potent at damaging plasma membranes, which may explain its lower hemolysis rate. Additionally, the surface charge and aggregation state of nanomaterials critically influence their in vitro cytotoxicity (Arivizo et al. 2010; Asharani et al. 2010; Liu et al. 2009). The surface charge of nanoparticles plays an especially important role in cell-nanoparticle interactions as cell membranes are charged as well. The charged surface of GO nanoparticles may allow it to interact with proteins present in the blood, which may result in hemolysis of RBCs. Another mechanism causing nanoparticle induced hemolysis is the generation of ROS (Yu et al. 2011). Oxidative stress can be involved in the toxic effects of graphene-based nanomaterials. Assessment of ROS production showed a toxic influence of GN, GO, and rGO on RBC. The interactions of the graphene with the RBC can lead to excessive ROS generation. A disruption of membrane functionality from an interaction between graphene platelets and the cell membrane ultimately causes damage to the cell due to oxidative stress.

In general, the hemolytic properties of graphene nanoplatelets are influenced by their size, shape, surface charge, and chemical groups on the surface (Yu et al. 2011).

Liao et al. (2011) reported, similarly to our study, hemolytic activity of graphene nanoplatelets towards RBC. However, in another study, pristine and functionalized graphene had no effect on hemolytic activity (Strojny et al. 2015).

Hemocompatibility assays are of crucial importance in evaluating potential biomedical applications of nanoparticles, as most of them require intravenous administration resulting in direct interaction of nanoparticles with RBC and different immune cells. In agreement with previous studies, we suggest that the method of production and modification of graphene plays a key role in its hemocompatibility and should be carefully and thoroughly considered before therapeutic application.

## Conclusions

GN, GO, and rGO incubated with chicken embryo RBC caused damage to the structure of RBC and induced dose-dependent hemolysis. Treatments with all forms of graphene led to structural damage of cell membranes and formation of knizocytes and echinocytes. However, there were significant differences between the negative impact of the studied graphene forms, indicating that hydrophobic, reduced graphene nanoparticles (GN and rGO) are more toxic than those of the hydrophilic, oxidized form (GO). Moreover, GN produced by physical method of exfoliation had a higher hemolytic activity compared to chemically produced rGO. Our findings showed that different forms of graphene, depending on methods of production and surface modification, have a different hemocompatibility. Thus, these factors should be carefully studied and considered before medical application.
